# Synthesis of Gemini-type imidazoline quaternary ammonium salt using by-product fatty acid as corrosion inhibitor for Q235 steel

**DOI:** 10.1038/s41598-024-64671-8

**Published:** 2024-06-15

**Authors:** Yuting Ma, Weijun Qi, Min Yu, Nengkun Huang, Ruiming Li, Jihuai Tan, Xinbao Zhu

**Affiliations:** 1 Jiangsu Co-Innovation Center of Efficient Processing and Utilization of Forest Resources, College of Chemical Engineering, Nanjing Forestry University, Nanjing, 210037 China; 2 School of Biological and Chemical Engineering, Zhejiang University of Science and Technology, Hangzhou, 310023 Zhejiang China; 3 Zhejiang Xinyuan Industrial Co., Ltd, Tonglu, 311500 Zhejiang China

**Keywords:** Corrosion inhibitor, Imidazoline, Electrochemistry, Adsorption, Environmental protection, Corrosion, Green chemistry

## Abstract

Gemini-type imidazoline quaternary ammonium salt is a new type of environmentally friendly corrosion inhibitor has been widely used in engineering materials. However, most of them are hazardous/toxic compounds derived from petroleum-based products, which did harm to environment. In this work, an environmentally friendly Gemini-shaped imidazoline quaternary ammonium salt corrosion inhibitor (G211) was synthesized using cheap fatty acid recycled from dimer acid industry as feedstock. The corrosion inhibition effects of G211 on Q235 steel in 1 M HCl solution were investigated through weight loss experiments, potential polarization curves, and alternating current impedance spectroscopy experiments. The results show that the inhibition rate of G211 as a mixed-type inhibitor is up to 94.4% and the concentration drop as low as 500 ppm at 25 ℃. The adsorption of G211 on Q235 surface follows Langmuir adsorption isothermal curve. The chemical composition of the Q235 steel surface was analyzed through scanning electron microscopy (SEM) and X-ray photoelectron spectroscopy (XPS). Furthermore, the possible corrosion inhibition mechanism of G211 on the surface of Q235 steel is proposed. This article not only presents an outstanding solution for safeguarding Q235 steel against corrosion but also introduces a feasible method for high-value utilization of monomer acid (MA).

## Introduction

Corrosion is a chemical/electrochemical reaction that occurs between a material’s surface and a corrosive medium, causing irreversible physical damage to the material especially for metals in acidic environment^1^. To date, several methods including utilization corrosion inhibitors^2^, the utilization of surface-modified cationic coatings^3^ and electrochemical means^4^ have been employed to address above issue. Among these, corrosion inhibitors such as imidazoline derivatives^5–7^, thiourea derivatives^8^, isoxazolium derivatives^9^, inorganic chromates^10^, and organic derivatives^11^were considered as economically efficient approaches, to enhance the metal corrosion resistance due to their outstanding adsorption properties and structural stability. However, the production of common corrosion inhibitors required petroleum-based products as raw materials, resulting in the harmful to the environment. Biomass as raw materials such as bagasse oil^12^, rice bran oil^13,14^, lignin-derivative ionic liquids^15^, wasted mango seeds^16^, wasted avocado oil^17^, rose fruit^18^has been explored to produce environmentally friendly corrosion inhibitors because they not only exhibit superior performances but also exert minimal pressure on the environment^19^. Nevertheless, the procedures for production of those corrosion inhibitors required tedious and complex extraction which still limited their large-scale commercial production.

Dimer acid is made of vegetable oils, which has been used in various fields such as coating, adhesive, plasticizer, as well as printing ink due to its non-toxic, cheap and biodegradable. In the production process, dimer acid was synthesized by the Diels–Alder reaction of polyunsaturated linoleic acid, however, low iodine value monomer acids (MA) including stearic acid, oleic acid, and palmitic acid, were reserved as waste acids. Traditionally, MA is used as feedstock to respectively produce cheap soap and biodiesel^20^, and the general consumption is hard to satisfy the dimer acid industry, resulting in a substantial backlog of MA. Additionally, because MA is a mixed acid, it is difficult to employ in cosmetics in consideration of the tedious separation process. Recently, Tan et al.^21,22^ developed two bio-plasticizers using MA as feedstock and attempted to serve as excellent alternatives to entirely replace toxic DOP, which efficient facilitated the valuable utilization of industrial waste monomer fatty acids. Additionally, Qi et al.^23^synthesized imidazoline using MA as a raw material and the resulting product was used to modify glucosides to create a biodegradable corrosion inhibitor for Q235 steel, where a corrosion inhibition rate could reach up to 96.8%. However, the complexity of this synthetic route still impeded the industry application. Gemini imidazoline quaternary salt is a new type of environmentally friendly corrosion inhibitor. This molecule contains more N atoms, has a larger surface area, and therefore may have a higher chance of coordinating adsorption with metal surfaces, potentially resulting in greater adsorption strength. Mohammad Mobin et al.^24^ synthesized an environmentally friendly cationic Gemini surfactant using *N*,*N*-dimethylhexadecylamine as the raw material. It can effectively inhibit the corrosion of mild steel (MS) in 1 M HCl. Theoretical calculations have also demonstrated that N, O atoms can form an adsorption film on the surface of carbon steel for corrosion protection. Ruby Aslam et al.^25^ synthesized a sugar-based *N*,*N*′-didodecyl-*N*,*N*′-digluconamideethylenediamine Gemini surfactant and used it together with KI as a corrosion inhibitor for mild steel in 3.5% NaCl solution. The results showed a synergistic inhibition effect between the two, with the highest inhibition rate reaching 97%. This represents a spontaneously occurring, strong, and stable corrosion inhibitor.

Herein drawing inspiration from the significant market demand for corrosion inhibitors, this study initiates with the utilization of a plethora of industrial by-product methylamine (MA) to overcome the challenges associated with synthesizing sustainable and cost-effective Gemini-type imidazoline quaternary ammonium salts. A specific derivative, designated as G211, is synthesized through a straightforward pathway, employing MA as the primary feedstock. This research harnessed MA as a biomass-derived material in conjunction with compounds, such as hydroxyethyl ethylenediamine, glycerol, and epichlorohydrin, to craft an environmentally friendly Gemini-type imidazoline quaternary ammonium salt, serving as a corrosion inhibitor for Q235 steel, structures were characterized in detail. Utilizing chemical and electrochemical methods, a comprehensive study on the corrosion inhibition capabilities of G211 on Q235 steel in 1 M HCl solution was conducted. The thermodynamic features of its interaction with the Q235 surface were deliberated, and the plausible corrosion inhibition mechanism was expounded through the application of SEM–EDS, XPS, and quantum chemical calculations. Empirical findings highlighted G211’s remarkable prowess in inhibiting corrosion, affirming its efficacy as a corrosion inhibitor for Q235 steel within acidic environments. This work not only introduced an exceptional solution for shielding Q235 steel against corrosion but also proposed a practical avenue for maximizing the value of MA, significantly unlocking its potential within industrial production.

## Experimental section

### Materials

Monomeric acid (wt%: palmitic acid 23.10, oleic acid 31.24, stearic acid 37.13, octadecadienoic acid 4.73, and arachidic acid 3.62) was obtained from Anhui Hongtai New Materials Co., Ltd. Hydroxyethyl ethylenediamine, epichlorohydrin (ECH), boric acid, xylene, and methanol were obtained from Shanghai Macklin Biochemical Co., Ltd. Q235 steel was obtained from Hefei Wenghe Metal Materials Co., Ltd. 1 M HCl solution was prepared from redistilled water and reagent grade HCl (37% wt).

Q235 steel is cut into 10 × 10 × 0.5 mm slats. Before the corrosion test, scrape the working surface with 800 and 1200 grade sandpaper, and then wash and degrease the surface iron filings with deionized water and acetone. The chemical composition of Q235 is given in Table 1.

**Table 1:** Chemical composition of Q235 steel.

Fe	C	Si	Mn	P	S	Cr	Ni	Al
Bal	0.012	0.001	0.001	0.011	0.008	0.01	0.001	0.006

### Synthesis of G211

G211 was synthesized through a three-step reaction, and the synthetic processes are depicted in Scheme I.

**Scheme I:**
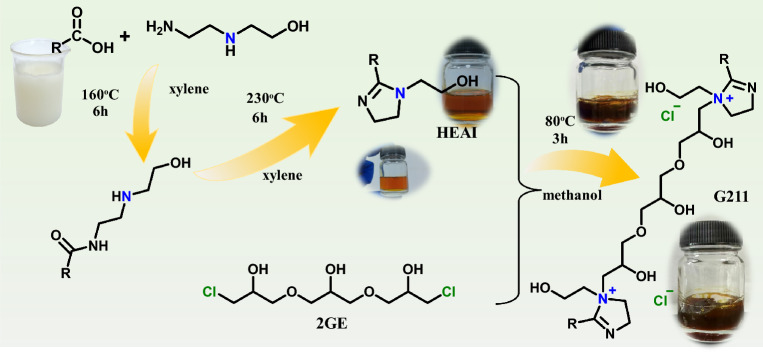
Synthesis route of G211.

Boric acid was selected as catalyst. First, MA (0.5 mol), hydroxyethyl ethylenediamine (0.7 mol) catalyst (0.8 g), xylene(65.03 g) were added to a three-necked flask equipped with a condenser and stirrer, heated into 160 ℃ for 6 h, the system was heated to 230 ℃ and it was held for 6 h. HEAI was obtained by distillation under reduced pressure to remove xylene and excess hydroxyethyl ethylenediamine. Subsequently, 0.5 mol 2GE and 15 ml methanol were added to the reactor. The system was kept at 85 ℃ for 3 h. G211 was obtained by distillation under reduced pressure to remove methanol.

### Measurement methods

The corrosion rate and corrosion inhibition performance of Q235 steel was evaluated by weight loss experiment.Q235 steel ,cut and dried, immersed in 1 M HCl solution at 25–65 ℃, with different concentrations of G211 corrosion inhibitor. After a 6 h exposure under these conditions, the alterations in the mass of the Q235 steel were recorded.The corrosion rate can be calculated using Eq. (1)^26^, and the corrosion inhibition rate can be calculated using Eq. (2)^27^.

1 $$ C_{R} = \frac{{W_{0} - W_{1} }}{A \times t} $$

2 $$ IE\% = \frac{{C_{R0} - C_{R1} }}{{C_{R0} }} \times 100\% $$

Conducting electrochemical tests utilizing the CS2350H three-electrode electro-chemical workstation to evaluate the corrosion inhibition effectiveness. The Q235 steel functions as the working electrode, whereas the platinum electrode and saturated calomel electrode serve as the auxiliary and reference electrodes, respectively. The test area is 1 cm^−2^. Potentiodynamic polarization curve (PDP) test was were obtained with a 1 mV/s scanning rate from − 1.0 to 0.2 V. The corrosion inhibition rate can be calculated using Eq. (3)^28^. Where I_corr,0_ and I_corr_ are corrosion current densities in solution without and with inhibitor, respectively.

Electrochemical impedance spectroscopy (EIS) tests were performed using a 10 mV AC disturbance to measure the amplitude, covering a frequency range from 100 kHz down to 0.05 Hz. And the corrosion inhibition rate can be calculated using Eq. (4)^29^. R_ct,0_ and R_ct_ are the charge transfer resistance values without and with inhibitor, respectively.

3 $$ IE\% = \frac{{I_{corr,0} - I_{corr} }}{{I_{corr,0} }} \times 100\% $$

4 $$ IE\% = \frac{{R_{ct} - R_{ct,0} }}{{R_{ct} }} \times 100\% $$

Applying the Langmuir adsorption isotherm model, as described in Eq. (5)^30^, the connection between the inhibitor’s adsorption capacity at equilibrium and its concentration was established. By employing the Langmuir isotherm equation, the Gibbs free energy of adsorption (∆G_ads_) was calculated via Eq. (6)^31^ after the K_ads_ have been calculated. And other thermodynamic parameters (∆H_ads_, ∆S_ads_) are calculated by Eq. (7)^32^.

5 $$ \frac{\theta }{1 - \theta } = K_{ads} C $$

6 $$ \Delta G_{ads} = - RTln\,(C_{{H_{2} O}} K_{ads} ) $$

7 $$ \log K = \left( { - logC_{{H_{2} O}} + \frac{{\Delta S_{ads} }}{2.303R}} \right) - \frac{{\Delta H_{ads} }}{2.303RT} $$

### Characterization

Fourier transform infrared (FT-IR) spectroscopy was performed utilizing a Bruker spectrophotometer (VERTEX 80 V) equipped with attenuated total reflection (ATR). The range of wavenumber was 4000-650 cm^−1^ and 32 scans per spectrum were collected with the resolution of four wavenumbers. ^1^H NMR spectroscopy was performed by a Bruker Biospin NMR apparatus (AVANCE III HD) with CDCl_3_ as solvent. SEM–EDS text was conducted with the FEI Quanta 200 scanning electron microscope, and XPS text was studied by a Shimadzu AXIS Ultra DLD X-ray photoelectron spectrometer with Al Kα (1486.6 eV) radiation. Before these text, Q235 steel samples were submerged in a 1 M HCl solution, with the addition of a 0 ppm and 500 ppm concentration of the G211 corrosion inhibitor. Afterwards, the samples were retrieved, and their surface were delicately cleaned and dried.

### Theoretical study

Considering that G211 can be ionized in solution, quaternary ammonium ion is used for calculation. After establishing the 3D configuration of the G211 cation. The initial configuration of the molecule was calculated using Gaussian 09W software and B3LYP/6-31G (d,p) base group by density functional theory (DFT) method, and the structure was optimized using water as solvent^33,34^. The results were visualized by Multiwfn^35^and VMD software.

## Results and discussion

### Structural characterizations

**Figure 1:**
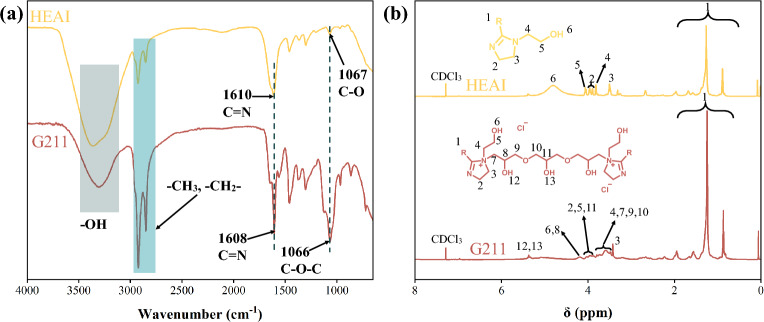
FT-IR, and ^1^H NMR spectrum of HEAI and G211.

The FT-IR spectra of MA, HEAI and G211 are shown in Fig. 1a. Neither the HEAI nor the G211 spectra exhibited the –COOH peak and the presence of the C=N^36^ bond at 1610 cm^−1^ in the spectrum of HEAI prove that we have successfully synthesized HEAI. In the G211 spectrum, the pronounced absorption peaks of C–O–C bond at 1066 cm^−1^ signifies the successful incorporation of 2GE into the HEAI structure, thereby confirming the synthesis of Gemini quaternary ammonium salts, what more, the absorption peak of C=N bond at 1608 cm^−1^ also appears in the G211 spectrum.

The ^1^H NMR spectra of the relevant compounds are depicted in Fig. 1b. In ^1^H NMR spectra, the –COOH peak was not observed in either the HEAI or the G211 spectra, and the appearance of the imidazoline ring (3.3, and 3.9 ppm) in HEAI spectrum implies the successful reaction between MA and hydroxyethyl ethylenediamine. In the G211 spectrum, the appearance of C–O–C and –CH– bond at 3.5, and 3.9 ppm implies the successful addition between HEAI and 2GE, and in G211 spectrum, it still shows the peak of imidazoline ring(3.3, and 3.9 ppm). These results demonstrated that G211 have been synthesized successfully.

### Corrosion inhibition performance

The corrosion rates and inhibition efficiencies on Q235 steel under various G211 corrosion inhibitor concentrations and temperatures through weight loss text are depicted in Table 2.

**Table 2:** The results of the weight loss method of Q235 at different temperatures and different concentrations of G211 inhibitor.

G211 Com (ppm)	Weight loss(mg)					IE(%)				
	25 ℃	35 ℃	45 ℃	55 ℃	65 ℃	25 ℃	35 ℃	45 ℃	55 ℃	65 ℃
Blank	6.3	13.7	24.5	40.2	80.8	–	–	–	–	–
100	0.9	1.0	1.0	1.6	2.4	85.71	92.70	95.92	96.02	97.03
200	0.7	0.9	0.9	1.4	2.2	88.89	93.43	96.33	96.52	97.28
300	0.6	0.5	0.8	0.9	1.7	90.48	96.35	96.73	97.76	97.90
400	0.5	0.4	0.8	0.9	1.3	92.06	97.08	96.73	97.76	98.39
500	0.4	0.4	0.7	0.8	0.7	93.65	97.08	97.14	98.01	99.13

G211 demonstrates effective corrosion inhibition properties across a wide range of concentrations and temperatures. As the concentration of G211 increases, the inhibition efficiency steadily improves, indicating that more G211 inhibitor molecules are adsorbed on the metal surface, reducing the contact area between Q235 and corrosive media such as acidic ions, thereby reducing the corrosion rate. It shows that Fe in the blank sample is more prone to electron loss, entering the solution as Fe^2+^ or Fe^3+^^37^, as the temperature rises, corrosion becomes more pronounced, leading to a significant increase in weight loss and an accelerated corrosion rate for Q235. However, when G211 corrosion inhibitor is introduced, the weight loss remains relatively stable. With increasing temperature, the inhibition efficiency gradually improves. This could be attributed to elevated temperatures enhancing the thermal motion of inhibitor molecules, facilitating the formation of coordination bonds between the metal surface and the inhibitor molecules. This results in strong inhibitor attachment to the metal surface, making it challenging to desorb once the bond is formed. Additionally, this observation suggests that the adsorption of G211 corrosion inhibitor on the metal surface primarily involves chemical adsorption, characterized by strong and irreversible bonding, which further confirms that G211 does not desorption from the metal surface as temperature increases^38^.

**Figure 2:**
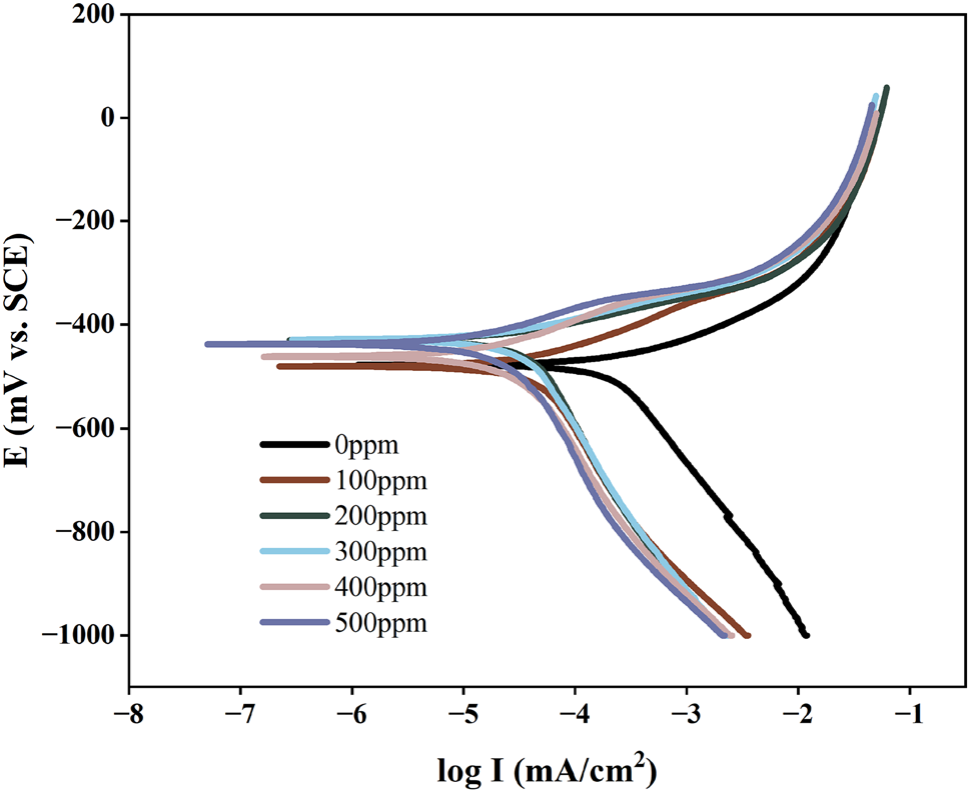
PDP test of Q235 steel in 1 M HCl solution with various concentrations of G211.

The outcomes from the PDP test shown in Fig. 2 reveal that following the inclusion of the G211 inhibitor, there is a noticeable shift towards lower current density in both the cathodic and anodic curves. This implies that G211 possesses the capability to inhibit the electrochemical reactions taking place at both the anode and cathode. Specifically, the cathodic curve shifts to the left while maintaining its original shape, signifying that the introduction of G211 impacts the reaction activity without modifying the underlying reaction mechanism of the metal electrodes^39^. Moreover, upon the addition of G211, the polarization curve shifts leftwards, signifying that even a small quantity of G211 can proficiently impede charge transfer, resulting in a reduction in current density. Additionally, the curve situated in the anodic region rapidly reaches a plateau region as the ‘potential increases’^40^, in this region, as the potential increases, the corrosion current density undergoes a steep ascent, and the inhibitor molecules desorb from the surface of Q235. Within the anodic area, as the polarization potential escalates, the PDP curve for G211-added solutions aligns almost parallel to the curve without G211, indicating a complete desorption of the inhibitor molecules^41^. This phenomenon characterizes interfacial inhibitors, wherein the molecules engage in a dynamic process of adsorption and desorption on the metal surface to safeguard it against corrosion. As the anodic potential intensifies, it facilitates the dissolution of the metal anode, consequently disturbing the adsorption–desorption equilibrium of the inhibitor molecules. When the rate of desorption exceeds that of adsorption, the metal’s Icorr increases. Moreover, with an escalation in the inhibitor concentration, the desorption potential shifts towards the anode, highlighting that a higher dosage of G211 can fortify a more enduring adsorption on Q235 steel.

Table 3 shown that following the incorporation of the G211 inhibitor, the range of Ecorr variation consistently remains below 85 mV^42^, which indicates that G211 operates as a mixed-type inhibitor. After adding G211 into the solution, noticeable changes occur in the slopes of both the cathodic and anodic Tafel curves, with a more pronounced alteration observed in the anodic slope. This indicates that upon introducing G211, adsorption occurs on the surface of Q235, effectively disrupting the reaction sites on the metal surface^43^, without altering the anodic reaction mechanism. Consequently, it reduces the current passing through the metal surface, thereby achieving the inhibitive effect. In the control sample, the Icorr reached 562.58 μA/cm^2^, signifying corrosion on the surface. Upon adding a concentration of 500 ppm, the Icorr decreased to 36.53 μA/cm^2^, significantly reducing the anodic dissolution. The results from the PDP test demonstrate that upon the addition of G211, inhibitor molecules adhere to the metal surface, occupying reaction sites and providing protection to the metal index.

**Table 3:** Electrochemical parameters of G211 obtained from polarization curves.

Dose (ppm)	E_coor_ (mV)	β_a_ (mV/dec)	β_c_ (mV/dec)	I_coor_ (μA/cm^2^)	IE (%)
0	− 477.83	118.24	− 286.19	562.58	–
100	− 477.34	88.84	− 281.35	55.31	90.17
200	− 494.54	91.87	− 291.06	49.71	91.16
300	− 460.58	78.35	− 263.33	44.63	92.07
400	− 461.61	83.05	− 286.02	43.29	92.31
500	− 478.40	86.12	− 219.77	36.53	93.51

**Figure 3:**
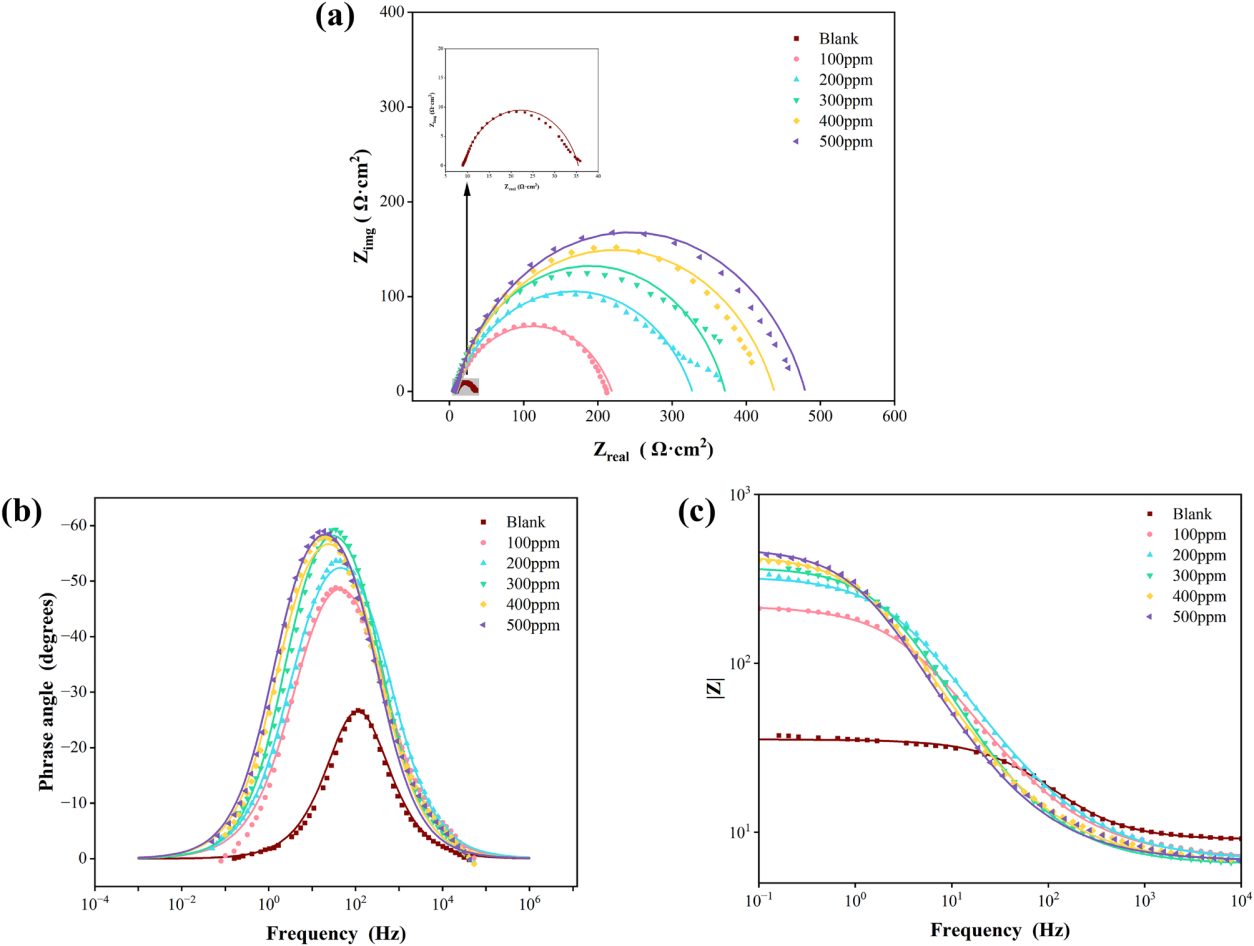
EIS test of Q235 steel in 1 M HCl solution with various concentrations of G211.

**Figure 4:**
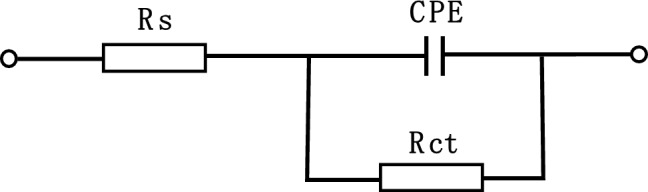
Equivalent circuit for Q235 steel in 1 M HCl solution.

The results of the EIS test for Q235 steel dipped in 1 M HCl solution are plotted using Nyquist and Bode graphs in the absence and presence of various concentrations of G211, as portrayed in Fig. 3. In Fig. 3a the radius of the semicircular curve depicted in the Nyquist plot rises in proportion to the concentration of G211 corrosion inhibitor. Upon reaching a concentration of 500 ppm, the impedance value attains to the max. The fitting outcome can be approximated as a distorted semicircle, suggesting that the impedance behavior of the Q235 steel electrode deviates from ideal behavior and exhibits a ‘dispersion effect’. This phenomenon is commonly attributed to the non-uniformity of active sites on the metal surface or disparities in conductivity. The results of the Bode plot are depicted in Fig. 3b and c, with the addition of G211 corrosion inhibitor, a time constant is observed, indicating that the electrochemical corrosion process is mainly controlled by the charge transfer step^44^ at the interface. It is highly likely that this inhibitor functions as an adsorption-type corrosion inhibitor, aligning with the outcomes obtained from the PDP test. Therefore, based on the Nyquist and Bode plots obtained from the experiment, Fig. 4 is drawn as the equivalent circuit of G211 corrosion inhibitor for Q235 steel in 1 M HCl solution, and it is fitted to the experimental data. The calculation results, obtained using Eq. (4), are presented in Table 4.

**Table4:** Fitting parameters and corrosion inhibition rate of different concentrations of G211.

Dose (ppm)	R_s_ (Ω cm^2^)	CPE		R_ct_ (Ω cm^2^)	EI (%)
		Y_0_ (S^n^/cm^2^Ω × 10^4^)	n		
0	9.034	4.113	0.7938	26.4	–
100	7.006	4.265	0.7324	211.9	87.54
200	6.854	3.285	0.7417	320.5	91.76
300	6.455	3.099	0.7993	365.0	92.77
400	6.732	3.931	0.7710	431.2	93.88
500	6.785	3.969	0.7862	472.8	94.42

Usually, the capacitive reactance arc that appears in the first quadrant is caused by the parallel connection of resistance and capacitance^45^. In the equivalent circuit, a constant phase element (CPE) is introduced to characterize the ‘dispersion effect’. Moreover, from the Nyquist plot, it can be seen that there is a resistance for solution in the electrolytic cell 5. Based on the above analysis, the equivalent circuit shown in Fig. 4 was drawn as the equivalent circuit formed between the G211 corrosion inhibitor and the Q235 metal surface, and it was fitted to the experimental data. As shown in Fig. 4, R_s_ is the solution resistance, R_ct_ is the charge transfer resistance, and CPE is the constant phase Angle element, which contains two parameters, Y_0_ and n, that is, Y_0_ is the parameter of CPE, and n is the dispersion.

Table 4 shows that for the G211 corrosion inhibitor, as the concentration increases, R_ct_ increases, indicating that more and more G211 corrosion inhibitor molecules replace the water molecules on the metal surface. The structure of the G211 corrosion inhibitor molecule contains two N^+^ ions, which facilitate electrostatic adsorption on the Q235 steel surface. With an increase in the number of molecules, the shielding effect intensifies, leading to a gradual rise in EI%. When the concentration of the corrosion inhibitor is 500 ppm, the corrosion inhibition efficiency reaches the optimal value of 94.42%. Generally, when there is a dispersion effect on the electrode surface, the n value is always between 0.5 and 1^46^, which is consistent with fitting results. It can also be seen from Table 4 that with the addition of G211, the value of Rs gradually decreases. It shows that the addition of G211 can prevent the surface corrosion of the electrode, protect its basic characteristics, and keep a good charge transfer, which also corresponds to the change of the EI%. The trend is in agreement with the results of the weight loss test and the PDP test.

### Langmuir adsorption isotherm

**Figure 5:**
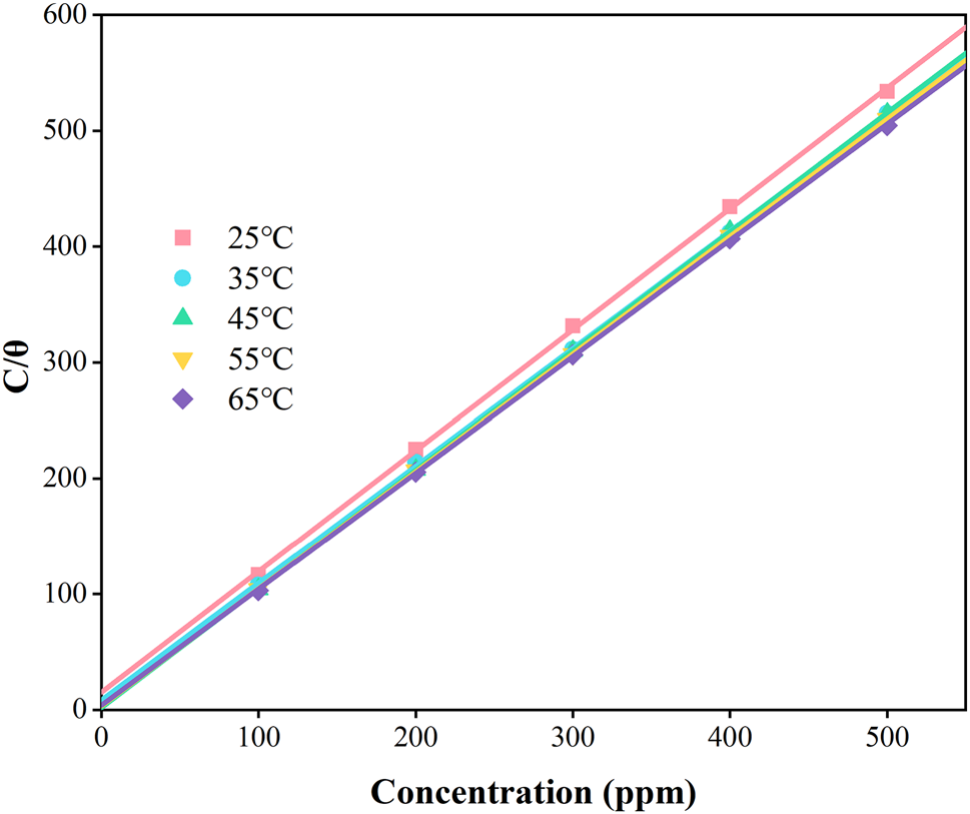
Langmuir isotherm adsorption for Q235 steel in 1 M HCl solution.

The Langmuir adsorption isotherm model was used to fit the data and it was found that the plot of C/θ versus C was linear, as shown in Fig. 5. At every temperature, the correlation coefficients (R_i_^2^) of both reached 0.999, indicating that the adsorption of G211 corrosion inhibitor on Q235 surface followed the Langmuir adsorption isotherm model.

Moreover, the calculated values of each ∆G_ads_ were showed in Table 5 and ∆Gads were all negative, indicating that the adsorption process of G211 corrosion inhibitor on Q235 steel surface was spontaneous. When the absolute value of ∆G_ads_ was less than 20 kJ/mol, it could be considered that the corrosion inhibitor molecules mainly physically adsorbed on the metal surface by electrostatic interaction. When the absolute value of ∆G_ads_ was greater than 40 kJ/mol, it could be considered that the corrosion inhibitor molecules were mainly chemically adsorbed on the metal surface by forming coordination bonds. The experimental ∆G_ads_ value falls within this range, suggesting that the adsorption of G211 on the Q235 surface is the synergistic outcome of two distinct adsorption modes.

**Table 5:** Thermodynamic adsorption parameters of G211 corrosion inhibitor on Q235 steel surface in 1 M HCl solution at different temperatures.

Temp (℃)	R^2^	Slope	K_ads_ × 10^4^ (M^−1^)	∆G_ads_ (kJ × mol^−1^)	∆H_ads_ (kJ × mol^−1^)	∆S_ads_ (J × mol^−1^ × K^−1^)
25	0.999	1.04	2.99	− 35.39	32.82	228.88
35	0.999	1.01	6.86	− 38.73		232.31
45	0.999	1.02	9.56	− 40.65		231.03
55	0.999	1.01	11.54	− 42.60		229.94
65	0.999	1.00	16.40	− 44.91		229.97

### Surface analysis

**Figure 6:**
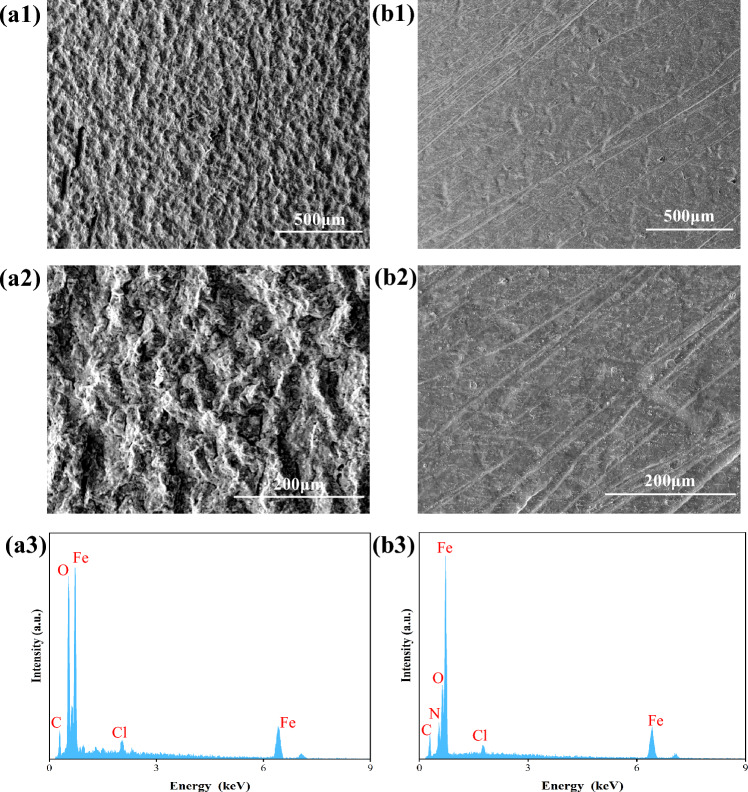
The SEM images and EDS results of Q235 steel after immersion of 7 days in 1 M HCl solution with (**a**) o ppm G211 and (**b**) 500 ppm G211.

The surface morphology changes of Q235 were observed by SEM test. Figure 6a1, a2 illustrates the surface of Q235 steel without the addition of G211 after soaking for 7 days, which has undergone serious corrosion, appearing extremely rough and loose, and the traces of sandpaper polishing have become blurred. On the contrary, in Fig. 6b1, b2, the surface of the sample with the addition of 500 ppm G211 corrosion inhibitor was smoother and retained relatively complete scratches. This indicates that G211 corrosion inhibitor can stably exert corrosion inhibition effect under acidic conditions, forming a dense protective film on the steel surface by adsorption, effectively hindering the corrosion of Q235 steel by acidic medium.

In addition, the results of EDS text are displayed in Fig. 6a3, b3, which show that the Q235 steel in the blank sample was continuously oxidized, and the content of Fe was reduced to 57.4%, while the Contet of O on the surface increased significantly to 35.0%. The condition was attributed to the oxidation of Fe to Fe^2+^ or Fe^3+^ in Q235 steel under acidic conditions, resulting in more stable oxides. Compared with the blank sample, the content of Fe on surface of Q235 steel remained at a relatively high level of 81.1% after adding the G211 corrosion inhibitor, and the O generation was suppressed to some extent, to 6.0%. Based on the above analysis, the confirmed test results from EIS and PDP demonstrate the successful adsorption of G211 molecules onto the surface of Q235 steel, thereby offering protection against oxidation^47,48^.

**Figure 7:**
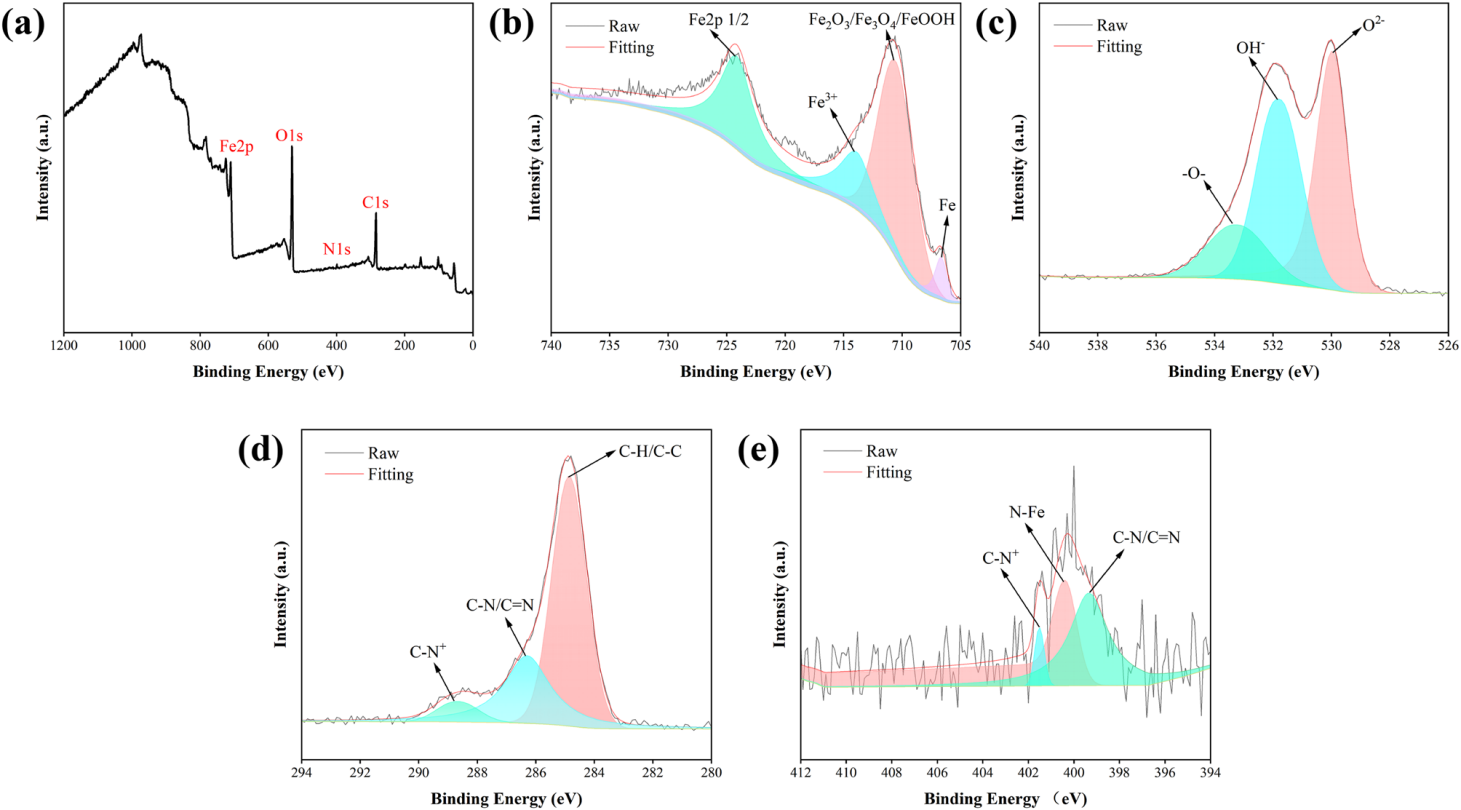
XPS spectra of Q235 steel after immersion of 48 h in 1 M HCl solution with 500 ppm G211: (**a**) wide spectra, (**b**) Fe 2p, (**c**) O 1 s, (**d**) C 1 s, (**e**) N 1 s.

Surface elemental analysis of Q235 steel at a G211 concentration of 500 ppm was further studied by XPS, and the results are displayed in Fig. 7a–e. Several elements, including C, N, O, and Fe, can be observed in the XPS spectrum. In wide spectra, the presence of N confirms that G211 has successfully adsorbed onto the surface of Q235 steel, aligning with the earlier test results. Four peaks appeared in the Fe 2p spectra, the component at 706.8 eV binding energy is attributed to the Fe in Q235 steel^30^ while at 710.8 eV may related to ferric oxides like Fe_2_O_3_, Fe_3_O_4_, or FeOOH, the peak at 714.2 eV binding energy might be associated with FeCl_3_ resulting from the testing environment, last peak at 724.4 eV was Fe 2p 1/2^49^. The O 1 s spectrum was deconvoluted into three peaks at 530.0, 531.9 and 533.3 eV, which correspond to O^2−^, OH^−^ and –O– bond respectively. The peaks at 530.0 and 531.9 eV are attributed to ferric oxides such as Fe_2_O_3_, Fe_3_O_4_, or FeOOH which are consistent with the Fe 2p spectrum, the –O– bond in the G211 molecule results in the appearance of a characteristic peak at 533.3 eV in the O 1 s spectrum, indicating that G211 has successfully adsorbed onto the metal surface. Three peaks emerged in the C 1 s spectrum at 288.7 eV (C–N^+^), 286.3 eV (C–N/C=N) and 284.8 eV (C–C), the peak at the binding energy of 288.7 and 286.3 eV can be thought to be caused by the C and N elements in the G211 molecule. The two peaks that appeared in the N 1 s spectrum, corresponding to 401.5 eV (C–N^+^), 400.4 eV (Fe–N) and 399.3 eV (C–N/C=N). The distinctive peak detected at 400.4 eV, signifying Fe–N, effectively confirms the existence of G211 inhibitor molecules on the surface of Q235. The results are consistent with the O 1 s and C 1 s spectrum, confirming the successful adsorption of G211 as a corrosion inhibitor on the surface of Q235 respectively^30,50^.

### Quantum-chemical calculation

**Figure 8:**
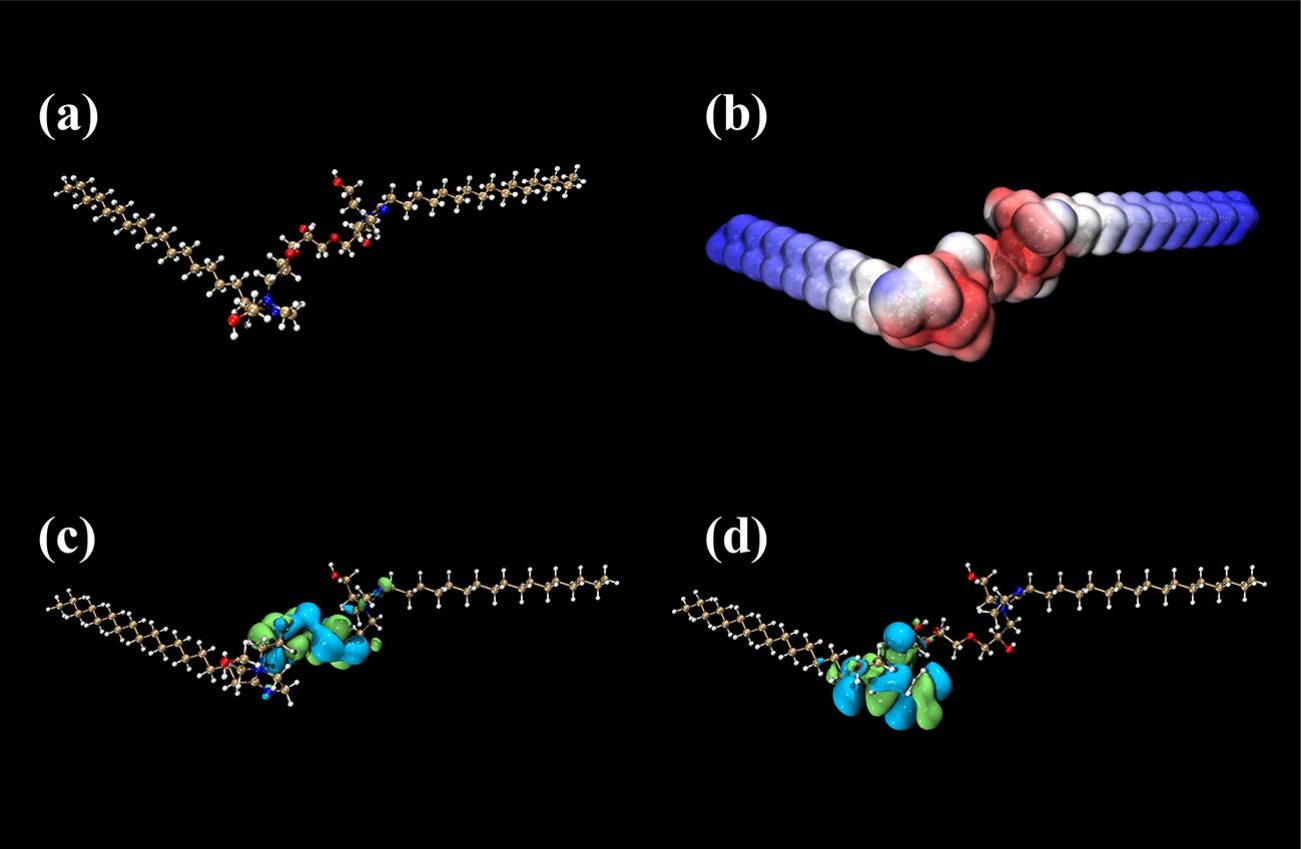
The results of quantum chemical calculations: (**a**) the optimized geometry configurations, (**b**) ESP image, (**c**) E_HOMO_ image and (**d**) E_LUMO_ image of G211 cation.

Density functional theory (DFT) is an important method to evaluate corrosion inhibition mechanism^51^. According to quantum chemical calculations, the results are shown in Fig. 8. Figure 8b shows the electrostatic potential (ESP) mappings on the van der Waals (vdW) surface of G211 quaternary ammonium cations, the positive pole region primarily concentrates on the red section. When G211 absorbs on Q235 steel, this particular area becomes susceptible as an electrophilic reaction attack site. The blue section, on the other hand, represents the negative pole region, which is more prone to attracting nucleophilic reagents for attack, when G211 absorbs, π electrons from Fe will attack this area. The charge density distribution of the molecular frontier orbitals of the G211 molecular compound shows that in the HOMO diagram, the charge density is most dense near the C–O–C bond, and the d orbital in the electronic orbital of Fe is not full, so the above-mentioned dense part of the charge distribution can provide π electrons for Fe, and there is a vacant d orbital in Fe, and the two can form a covalent bond. In the LUMO diagram, the charge density is mainly concentrated near N=C on the imidazoline ring, which can accept electrons from the Fe surface and form a feedback bond^49^. The results from the calculations of HOMO and LUMO align with the results obtained from ESP analysis. As a result, the corrosion inhibitor is adsorbed on the surface of Q235 steel, achieving the purpose of inhibiting corrosion^44,52^.

### Possible corrosion inhibition mechanisms

**Figure 9:**
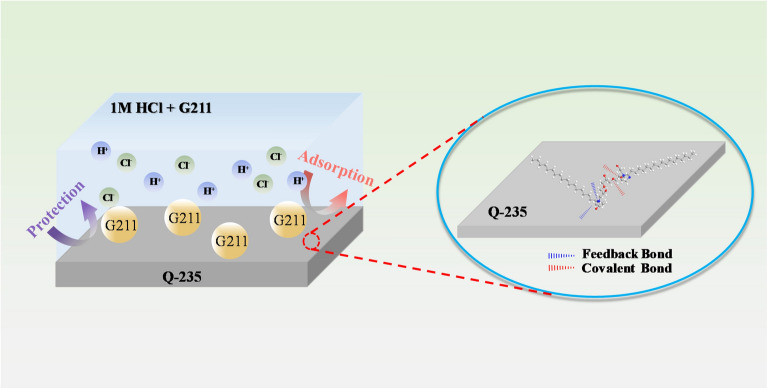
The possible corrosion inhibition mechanism of G211 corrosion inhibitor.

Based on the results of electrochemical tests, surface morphology elemental analysis, and quantum calculations, the possible corrosion inhibition mechanism of G211 is discussed in this work, which is displayed in Fig. 9. It forms a protective film by adsorbing and bonding with Fe atoms, isolating the corrosive substances from the metal surface. This isolation impedes anodic dissolution and suppresses the deposition of hydrogen at the cathode. The adsorption of G211 onto the Q235 surface primarily occurs due to the existence of lone pairs of electrons in N and O atoms, interacting with the three-dimensional unoccupied orbitals in Fe. Additionally, the antibonding orbitals in G211 form feedback bonds with electrons in Fe. The bicyclic imidazoline ring structure in G211 further facilitates central adsorption. The solution environment contains a significant amount of Cl^−^. These ions can adsorb onto the iron surface, forming (FeCl^−^) and generating a negative surface charge^49^, creating a negative surface charge. This promotes the physical adsorption of G211 cations onto the metal surface. Additionally, the structure of long-chain alkyls can hinder water molecules from coming into contact with the metal, thereby achieving the corrosion inhibition purpose.

## Conclusion

In this work, an environmentally friendly Gemini-shaped imidazoline quaternary ammonium salt G211 was synthesized using cheap MA, a by-product from dimer acid industry, as feedstock and as a corrosion inhibitor for Q235 in 1 M HCl solution. The results of the corrosion inhibition performance tests by chemical and electrochemical methods showed that the corrosion inhibition effect increased with the increase of G211 concentration in the solution. At a 500 ppm concentration, the corrosion inhibition efficiency at room temperature reached 94.4%. Moreover, the adsorption of G211 on the Q235 surface followed the Langmuir adsorption isotherm curve. According to the thermodynamic parameters, the value of ∆Gads indicated that G211 adsorbed on the metal surface by physical and chemical adsorption. In addition, findings from SEM–EDS and XPS tests have conclusively shown that G211 efficiently adsorbed to the surface of Q235, providing protection against corrosion. The quantum calculation results showed that the imidazoline ring and C–O–C bond were the active sites of G211, and they adsorbed on the metal surface by providing and accepting electrons from the Fe atoms. Considering the good performance of G211, MA exhibited potential as a raw material for the production of corrosion inhibitors.

## Data Availability

All data are included in the manuscript.
